# The Comparative Clinical Performance of Four SARS-CoV-2 Rapid Antigen Tests and Their Correlation to Infectivity In Vitro

**DOI:** 10.3390/jcm10020328

**Published:** 2021-01-17

**Authors:** Niko Kohmer, Tuna Toptan, Christiane Pallas, Onur Karaca, Annika Pfeiffer, Sandra Westhaus, Marek Widera, Annemarie Berger, Sebastian Hoehl, Martin Kammel, Sandra Ciesek, Holger F. Rabenau

**Affiliations:** 1 Institute for Medical Virology, University Hospital, Goethe University Frankfurt, 60596 Frankfurt, Germany; niko.kohmer@kgu.de (N.K.); Tuna.ToptanGrabmair@kgu.de (T.T.); christiane.pallas@kgu.de (C.P.); onurkaraca@hotmail.de (O.K.); s5936852@stud.uni-frankfurt.de (A.P.); sandra.westhaus@kgu.de (S.W.); marek.widera@kgu.de (M.W.); Annemarie.Berger@kgu.de (A.B.); sebastian.hoehl@kgu.de (S.H.); 2 Institut fuer Qualitaetssicherung in der Virusdiagnostik-IQVD der GmbH, 14129 Berlin, Germany; m.kammel@iqvd.de; 3 INSTAND Gesellschaft zur Foerderung der Qualitaetssicherung in Medizinischen Laboratorien e.V., 40223 Duesseldorf, Germany; 4 German Centre for Infection Research, External Partner Site, 60323 Frankfurt, Germany; 5 Fraunhofer Institute for Molecular Biology and Applied Ecology (IME), Branch Translational Medicine and Pharmacology, 60596 Frankfurt, Germany

**Keywords:** SARS-CoV-2, Ag-RDT, POCT, PCR, cell culture, infectivity

## Abstract

Due to globally rising numbers of severe acute respiratory syndrome coronavirus 2 (SARS-CoV-2) infections, resources for real-time reverse-transcription polymerase chain reaction (rRT-PCR)-based testing have been exhausted. In order to meet the demands of testing and reduce transmission, SARS-CoV-2 antigen-detecting rapid diagnostic tests (Ag-RDTs) are being considered. These tests are fast, inexpensive, and simple to use, but whether they detect potentially infectious cases has not been well studied. We evaluated three lateral flow assays (RIDA^®^QUICK SARS-CoV-2 Antigen (R-Biopharm), SARS-CoV-2 Rapid Antigen Test (Roche)), and NADAL^®^ COVID-19 Ag Test (Nal von Minden GmbH, Regensburg, Germany) and one microfluidic immunofluorescence assay (SARS-CoV-2 Ag Test (LumiraDx GmbH, Cologne, Germany)) using 100 clinical samples. Diagnostic rRT-PCR and cell culture testing as a marker for infectivity were performed in parallel. The overall Ag-RDT sensitivity for rRT-PCR-positive samples ranged from 24.3% to 50%. However, for samples with a viral load of more than 6 log_10_ RNA copies/mL (22/100), typically seen in infectious individuals, Ag-RDT positivity was between 81.8% and 100%. Only 51.6% (33/64) of the rRT-PCR-positive samples were infectious in cell culture. In contrast, three Ag-RDTs demonstrated a more significant correlation with cell culture infectivity (61.8–82.4%). Our findings suggest that large-scale SARS-CoV-2 Ag-RDT-based testing can be considered for detecting potentially infective individuals and reducing the virus spread.

## 1. Introduction

Since the discovery of SARS-CoV-2, the rRT-PCR-based diagnostic testing of respiratory specimens has been considered the gold standard [1]. The early detection of new infections impacts patient management and the control of transmission; thus, rapid and easy-to-use diagnostic assays, ideally eligible for point-of-care testing (POCT), are needed [2]. As of mid-December 2020, confirmed SARS-CoV-2 infections exceeded 70 million worldwide [3], and new cases continue to surge. Consequently, shortages of PCR kits and related consumables have emerged as major constraints in testing. Therefore, in settings where resources have become scarce, diagnostic rRT-PCR testing needs to be prioritized and reserved for critically ill patients or hospital admissions. Additionally, due to economic and logistic challenges and prolonged turnaround times, rRT-PCR-based testing may not be suitable for the screening of potentially infectious individuals such as visitors to nursing homes.

For these reasons, SARS-CoV-2 Ag-RDTs are being considered as a complement to PCR-based testing [4]. They are commonly based on the lateral flow principle and also work with respiratory specimens such as nasopharyngeal swabs, but, in contrast to PCR-based testing, they require no special training and generate results in a few minutes. Numerous commercial assays are now available [5], but there are limited data on their clinical performance. Based on previous studies, the sensitivity of SARS-CoV-2 Ag-RDTs range between 22.9% and 93.9% when compared to rRT-PCR [6,7,8,9,10,11,12,13,14]. Apart from sensitivity, it is important that such tests identify potentially contagious individuals to reduce SARS-CoV-2 transmission. Ag-RDTs and commonly used rRT-PCR-based assays detect the viral peptides and RNA of a fully formed infectious particle, respectively, as well as the non-infectious break-down products. Hence, these tests do not differentiate between infectious and non-infectious samples. Alternatively, subgenomic SARS-CoV-2 mRNA, generated via discontinuous transcription in the infected cells, can serve as a marker for actively replicating virus [15]. Subgenomic, but not total RNA detection in clinical samples, has demonstrated a better correlation with cell culture testing. Monitoring SARS-CoV-2 infectivity in cell culture may therefore be more informative to determine the infectiousness of a patient and potential transmission than standard rRT-PCR, which can reflect the persistence of non-replicating viral RNA [16,17]. Cell culture testing, however, is laborious and can only be performed in a BSL-3 laboratory by experienced personnel. AgRDTs, on the other hand, are affordable, scalable, and easy-to-use emerging diagnostic tools and thus have the potential to play a significant role in guiding patient management, public health interventions, and disease surveillance.

The objective of this study was to evaluate the clinical performance of three lateral flow assays and one microfluidic immunofluorescence assay, and the manufacturers’ prescribed lysis buffers for their ability to inactivate SARS-CoV-2. In parallel, all clinical samples were subjected to diagnostic rRT-PCR and positive samples were further subjected to cell-culture-based testing to provide a more thorough correlation analysis between each diagnostic assay platform.

## 2. Materials and Methods

### 2.1. rRT-PCR Analysis of Clinical Swab Samples and Intracellular RNA from Infected Cells

Dry nasopharyngeal swabs were taken from 100 individuals from shared living facilities for screening purposes regardless of their clinical symptoms and then suspended in 2 mL of phosphate-buffered saline (PBS) and incubated for 5 min. A total of 500 µL of the swab dilution was mixed with PCR lysis buffer (1:1 ratio) then transferred to barcoded tubes and subjected to rRT-PCR-analysis on the Cobas 6800 system (Roche Diagnostics International AG, Rotkreuz, Switzerland) system. The Cobas SARS-CoV-2 master mix was supplemented with an internal RNA control and primer–probe sets targeting ORF1 and E-gene according to the manufacturer’s protocol.

Intracellular RNA was isolated using RLT buffer (Qiagen, Hilden, Germany) and the RNeasy 96 HT Kit (Qiagen, Hilden, Germany) according to the manufacturer’s instructions. Subsequently, one-step rRT-PCR was performed on a CFX96 Real-Time System, a C1000 Touch Thermal Cycler, and the LightCycler^®^ Multiplex RNA Virus Master (Roche Diagnostics GmbH, Mannheim, Germany), as described elsewhere [18]. To detect SARS-CoV-2 sgRNA8, the following primer pair and probe were used: sgRNA8-F: 5’-ACCAACTTTCGATCTCTTGTAGA-3′; sgRNA8-R: 5′-GCTCACAAGTAGCGAGTGTT-3′; sgRNA8-P: 5′-CTGTTCTCTAAACGAACATGAAAATTATTC-3′ (HEX-BHQ2). Concomitantly, an input control targeting the human RNaseP gene (RPP30) was used in the multiplex to monitor the input of human nucleic acids (RPP30-F: 5′-AGATTTGGACCTGCGAGCG-3′; RPP30-R: 5′- GAGCGGCTGTCTCCACAAGT-3′; RPP30-P: 5′-TTCTGACCTGAAGGCTCTGCGCG-3′(Cy5-BHQ3)) [19].

### 2.2. Defining an rRT-PCR CT-Value Range for Potential Infectious Samples

Three quantitative comparison samples containing 10^5^, 10^6^, and 10^7^ SARS-CoV-2 (BetaCoV/Munich/ChVir984/2020) RNA copies/mL were used to generate a standard curve and to calculate the viral RNA copies/mL (Table S1 and Figure S1). The comparison samples were provided by INSTAND e.V. In total, 10 aliquots of each suspension were tested on two different days (5 aliquots/day) to verify the intra- and inter-assay reproducibility.

### 2.3. Chemical Inactivation of Cell Culture Supernatants

To test the lysis buffers included in the respective Ag-RDT kits, SARS-CoV-2 containing supernatant was mixed 1:1 with the respective lysis buffers and incubated for 10 min at ambient temperature. The mixture was further diluted and added to Caco-2 cells, as described previously [20]. The RNA from the infected cells was then used to assess the effectiveness of the inactivation.

### 2.4. Antigen-Detecting Rapid Diagnostic Tests (Ag-RDTs)

Aliquots of specimen-swab dilutions in PBS were tested within 24 h using four different SARS-CoV-2 Ag-RDTs, including three lateral flow assays and one microfluidic immunofluorescence assay: RIDA^®^QUICK SARS-CoV-2 Antigen (R-Biopharm AG, Darmstadt, Germany), SARS-CoV-2 Rapid Antigen Test (Roche Diagnostics GmbH, Mannheim, Germany), NADAL^®^ COVID-19 Ag Test (test cassette) (Nal von Minden GmbH, Regensburg, Germany), and the SARS-CoV-2 Ag Test on the LumiraDx™ Platform (LumiraDx GmbH, Cologne, Germany). All the assays were performed according to the manufacturers’ protocol, with some modifications to allow parallel testing using different platforms. An important difference is that we suspended the specimen swabs in 2 mL of PBS to allow cell culture (500 µL), rRT-PCR (500 µL) testing along with the Ag-RDTs (~800 µL for 4 tests) prior to testing. Due to limited sample volumes, the reagent amounts were adjusted accordingly (Table S2). In brief, 50 µL of sample and 50 µL of reagents A and B, respectively, were used for the R-Biopharm test; 100 µL of sample volume and 100 µL of each reagent volume were used for the Roche, Nal von Minden, and LumiraDx tests. This dilution ratio was lower than the manufacturer’s recommendations, which may have impacted the degree of virus inactivation. For lateral flow assays, the results were read visually and documented by three different individuals, and the majority consensus was chosen as the final test result. The average duration of the assays (excluding hands-on time) was 16 min (LumiraDx test: 12 min; the Nal von Minden test: 20 min).

### 2.5. Cell Culture and Detection of Infectious Virus

Caco-2 cells (human colon carcinoma cells) were obtained from DSMZ (Braunschweig, Germany, no.: ACC 169), differentiated by serial passaging, and selected for permissiveness for virus infection. The cells were maintained in Minimum Essential Medium (MEM) supplemented with 10% fetal calf serum (FCS, Sigma-Aldrich; St. Louis, MO, USA), 100 IU/mL of penicillin, and 100 g/mL of streptomycin. All the culture reagents were purchased from Sigma-Aldrich. Of the swab dilution, 500 µL was mixed with 1.5 mL of MEM containing 1% FCS, 7.5 µg/mL Amphotericin B (Sigma-Aldrich), and 0.1 mg/mL Primocin (InvivoGen; San Diego, CA, USA) and cultivated with Caco-2 cells seeded in 5.5 cm^2^ culture tubes. The cytopathogenic effect (CPE) was assessed daily, if possible, for up to seven days or until cell lysis occurred.

### 2.6. Statistical Analysis

The agreement between the Ag-RDTs and the cell culture results was evaluated using Cohen’s weighted kappa index (K value) [21]. K value interpretations were categorized as follows: <0.20 is poor, 0.21–0.40 is fair, 0.41–0.60 is moderate agreement, 0.61–0.80 is substantial agreement, and 0.81–1.00 is almost perfect agreement [22]. Clopper–Pearson confidence intervals for sensitivity/specificity were calculated using MedCalc (MedCalc Software, Ostend, Belgium).

Logistic regression analyses were implemented using Python in PyMC3, as previously described [23]. In brief, a default NUTS sampler with 25k samples, 5k tuning steps, and an acceptance probability of 0.95 was used. The program is described using the following pseudocode, where “db50” and “db95” are the decision boundaries for the concentration at which the test returns a positive result with 50% and 95% probability, respectively: x ← data [“log10 RNA copies”], y ← data [“test result”], α ← Normal (0, 15), β ← Normal (0, 15), log_odds ← α + βx, θ ← sigmoid (log_odds), y ← Bernoulli (θ).

## 3. Results

A total of 100 nasopharyngeal swab samples were collected from individuals living in a shared facility regardless of their infection status during a two-week period in November 2020 and were then processed and analyzed by rRT-PCR for SARS-CoV-2. A total of 74 samples tested positive, with primers targeting the ORF1 gene (Table S3). The cycle threshold (CT) values ranged between 22.13 and 36.46, corresponding to 7.1 to 2.73 log_10_ RNA copies/mL. In parallel, the samples were subjected to Ag-RDTs using four different assays. The overall detection sensitivity ranged between 24.3% and 50% for the rRT-PCR-positive (*n =* 74) samples (Table 1). All the tests were performed with a high specificity for the rRT-PCR-negative specimens (*n =* 26) (96.2–100%) (Table 1).

**Figure 1:**
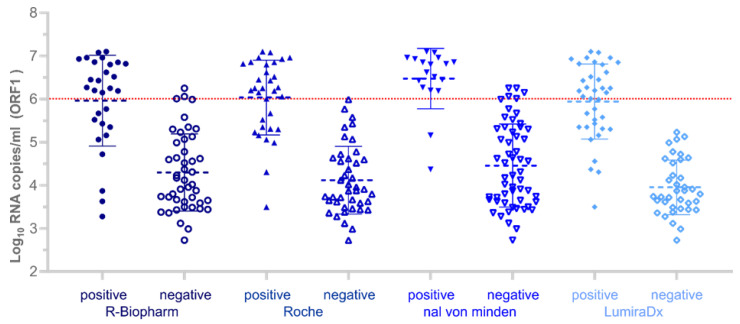
Antigen test analysis results for rRT-PCR-positive samples. Positive (filled data point symbols) and negative (empty data point symbols) Ag-RDT results and corresponding log_10_ RNA copies/mL for the ORF1 gene including mean and standard deviation bars for each test (*n* = 74). The dotted horizontal line in red indicates the literature-based hypothetical threshold for potential infectivity (6 log_10_ RNA copies/mL).

In terms of sensitivity within the range of potentially infectious samples (≥6 log_10_ RNA copies/mL), the LumiraDx and Roche tests demonstrated the highest sensitivity of 100%, the R-Biopharm showed a 85.7% sensitivity, and the Nal von Minden showed a 76.2% sensitivity. The distribution of SARS-CoV-2 RNA copies in the test samples across all the Ag-RDTs products is shown in Figure 1.

Regarding the comparative analysis of different Ag-RDTs with respect to their detection limits, we trained a binary logistic regression model as described previously [23] and performed an analysis for each Ag-RDT to determine the RNA concentrations at which the test gives a positive result with 50% and 95% probability (Table 2 and Figure S2). In this training model, LumiraDx achieved a positive test with a 95% probability at 5.979 log_10_ RNA copies/mL, which is considered to be the potential infectivity threshold.

**Figure 2:**
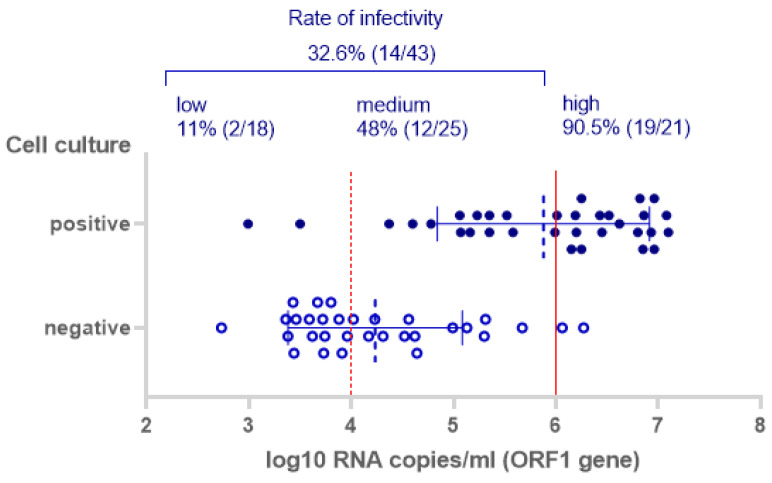
Cell culture results in correlation with the log_10_ RNA copies/mL of the ORF1 gene rRT-PCR reactive samples including mean and standard deviation bars (*n* = 64, 10 cytotoxic samples excluded).

For samples within the estimated infectious range for the ORF1 gene (>6 log_10_ RNA copies/mL), the cell culture test infectivity was 90.5% (19/21). However, for a fraction of samples with a lower viral load than the hypothetical threshold of potential infectivity, the cell culture test was also positive: 32.6% (14/43, excluding 10 cytotoxic samples). The cell culture results in correlation with the log_10_ RNA copies/mL of the ORF1 gene-reactive samples are shown in Figure 2.

The sensitivity and specificity of the examined SARS-CoV-2 Ag-RDT in correlation with cell culture testing are shown in Table 3. One additional ORF1 gene-negative and E-gene and cell culture test-positive sample (Sample 75) was included in the following analyses. The LumiraDx test demonstrated the highest sensitivity of 82.4% (65.5–93.2% 95% CI), followed by the Roche test with 70.6% (52.5–84.9%, 95% CI), the R-Biopharm test with 61.8% (43.6–77.8%, 95% CI), and the Nal von Minden test with 50% (32.4–67.6%, 95% CI). The Nal von Minden test showed the highest specificity of 96.8% (83.3–99.9%, 95% CI), followed by the R-Biopharm test with 93.6% (78.6–99.2%, 95% CI), and the Roche and LumiraDx test(s) both with 77.4% (58.9–90.4%, 95% CI).

Cohen’s weighted kappa coefficient between the Ag-RDTs and cell culture results showed a moderate agreement with a kappa for the LumiraDx test of 0.599 > R-Biopharm test of 0.545 > Roche test of 0.478 > Nal von Minden test of 0.457 (Table 4 and Tables S4–S7).

Regarding specificity testing of the rRT-PCR-negative samples, only the R-Biopharm test generated one false-positive result (ORF1 and E-gene negative). The other examined Ag-RDTs showed a specificity of 100%.

**Figure 3:**
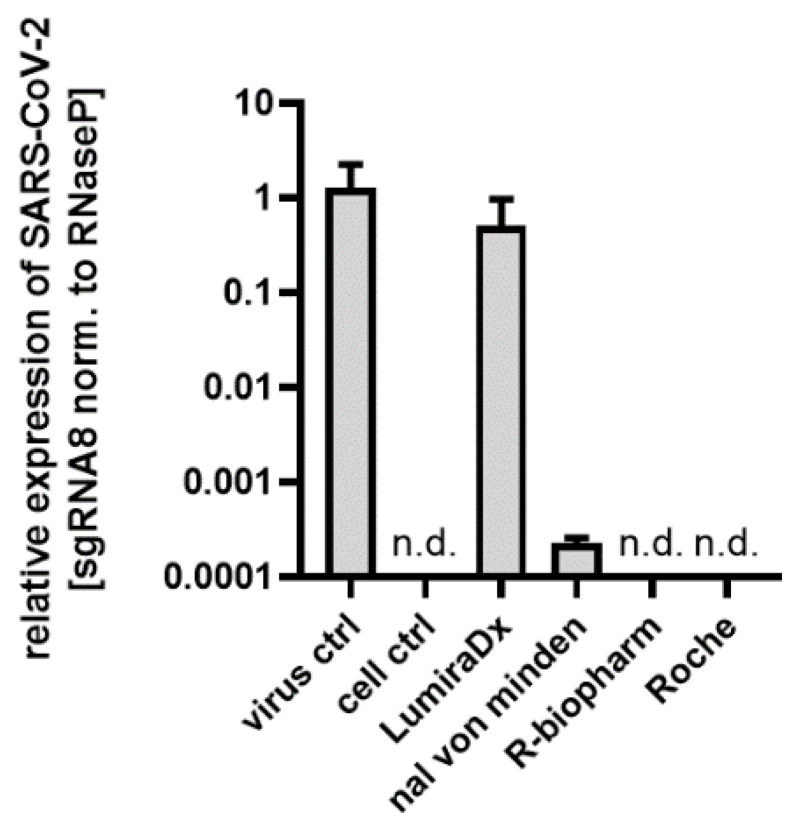
Detection and relative quantification of SARS-CoV-2 subgenomic mRNA isoforms in infected Caco2 cells. SARS-CoV-2 sgRNA8 mRNA levels were normalized to human RNaseP.

Furthermore, we evaluated the manufacturer’s prescribed lysis buffers for their ability to inactivate SARS-CoV-2, since handling infectious specimen material can be another source of viral transmission in POCT settings. For this purpose, a virus stock prepared in cell culture was treated with the same volume of lysis buffer and used to infect susceptible cells. In order to monitor virus replication, we isolated the cellular and viral RNA of the infected cells and performed an rRT-PCR assay targeting the SARS-CoV-2 subgenomic RNA 8. Surprisingly, we found that the lysis buffer for the LumiraDx test was not able to provide efficient virus inactivation properties, while all other three buffers resulted in a reduction in infectivity by several log levels (Figure 3). It should be noted that to be consistent throughout this study, aliquots of the swab dilutions and virus stock were diluted at a 1:1 ratio with the lysis buffer.

## 4. Discussion

The relatively poor performance of SARS-CoV-2 Ag-RDTs when testing clinical samples not differentiated by high versus low RNA concentration has already been described in different studies [10,11,12]. Ag-RDT sensitivity increases when testing samples with higher RNA or virus concentrations, which is likely to be during the pre-symptomatic and early phases of the infection [24]. Although the correlation between viral load and transmissibility is not entirely clear, several studies showed that samples with higher viral loads of ≥6 log_10_ RNA copies/mL were likely to correlate with infectivity in cell culture models [15,25,26,27,28]. We, and others, showed that Ag-RDTs, although less sensitive, align better with cell culture-based testing for infectivity than rRT-PCRs [14,25,29,30,31].

When rRT-PCR was conducted in our study, we primarily focused on the ORF1 gene as the target, because similar CT-values were generated when compared to the assay-specific E-gene tested in parallel (Table S3). But we used ORF1 values for comparative analysis since 3 samples (<6 log_10_ RNA copies/mL (ORF1 gene)) were negative with E-gene PCR, probably due to point mutations in the primer–probe binding sites within these isolates. With naturally emerging mutations within the SARS-CoV-2 genome, detection using Ag-RDTs can be impaired as well. Most of these tests detect the SARS-CoV-2 nucleoprotein; however, their performance should be regularly monitored as new missense mutations emerge within the detection region.

We determined that the sensitivity of the examined Ag-RDTs for the SARS-CoV-2 rRT-PCR reactive samples within the standardized potential infectious range for the ORF1 gene reactive samples was 76.2% (the Nal von Minden test), with a potential of up to 100% (the Roche and LumiraDx tests). Notably, more than 30% of the samples, despite having a relatively low viral load [<6 log_10_ RNA copies/mL (ORF1 gene)], still tested positive in cell culture. Although there is no direct evidence for the correlation between infectivity in cell culture and virus transmissibility in humans, the detection of infectious virus correlated with the communicable period in an animal model (Syrian gold hamster) [32] and is recognized as a marker of infectivity. It is unknown whether individuals with lower viral loads (<6 log_10_ RNA copies/mL (ORF1 gene)) that are positively tested in cell culture contribute to virus transmission. It is also possible that cell culture-based testing is too sensitive as a surrogate for transmission among humans. Susceptibility of the cell line used might influence the outcome as well. For samples containing ≥ 6 log_10_ RNA copies/mL, three out of four tests examined in our study met the minimum WHO performance requirements for the use of SARS-CoV-2 Ag-RDTs of ≥80% sensitivity and ≥97% specificity when compared to a nucleic acid amplification test as a reference assay [24].

In line with our results, previous studies analyzing the performance of different antigen tests reported a consistently high specificity but a broad spectrum of sensitivity that seems to be lower than the sensitivity range reported by the manufacturers [6,7,8,9,10,11,12,13,14]. These differences might be due to multiple factors, such as the moment of testing in the infection phase, the cohort size, the sampling site, the specimen quality, and the handling and preparation, or related to the distribution of CT-values using non-standardized rRT-PCR. The microfluidic immunofluorescence-based LumiraDx test demonstrated the highest sensitivity of 50% and 82.4% when compared to rRT-PCR and cell culture infectivity, respectively. The microfluidic system is also less vulnerable to subjective visual interpretation in terms of sample readout; however, the need for an electronic reader, processing one sample at a time, could be a limiting factor in the case of large sampling sizes. In the course of this study, we found that, in contrast to the other kits, the LumiraDx Lysis buffer did not have a sufficient inactivating activity, which may be due to the adapted dilution ratio of clinical samples and virus stock with the lysis buffer throughout this study. In the direct comparison of the methods, the dilutions must also be taken into account, which may have a direct influence on the CT values. The differences in Ag-RDT sensitivity observed in our study compared to the manufacturer’s specifications are possibly due to sample collection in the later phase of infection, as specimens were collected regardless of the individual’s infection status.

Regarding the observation that the R-Biopharm test examined in this study generated one false-positive result when compared to the gold standard—an rRT-PCR-negative tested sample—a certain degree of false positivity is common in diagnostic tests and requires an rRT-PCR validation. We recently reported a 0.15% false-positivity rate in a study comprising more than 10,000 home tests performed by school teachers [33], which underscores the importance of an rRT-PCR confirmation test at an individual level by also considering the prevalence of the disease [34]. The study by Corman et al. also observed a single cross-reactivity with Influenza A virus when using the predated R-Biopharm product, though no direct link to any specific respiratory agent could be found [23]. However, the marketed R-Biopharm product did not show any cross-reactivity with Influenza A or any other respiratory agents in the later analysis [14]. These results suggest that rigorous analysis for sensitivity and specificity should be performed prior to the commercialization of the kits.

Our study has clear limitations including limited sample size. Therefore, we were not able to perform any correlation analysis of demographic factors and symptoms within this group of samples. The major strength of this work is the detailed analysis of the clinical samples in the cell culture for infectivity. Correlation between rapid antigen testing and infectivity is an important indication of its clinical utility in controlling virus transmission.

The clinical sensitivity within the potential infectious range and moderate agreement with cell culture, including the above-mentioned limitation, allow large-scale SARS-CoV-2 Ag-RDTs-based testing to be considered as a surrogate marker for identifying potentially infective individuals in a population, reducing the spread to others. This strategy may be particularly effective when the tests are used frequently [35]. Focusing on the clinical sensitivity within the potential infectious range is a more practicable approach than focusing just on the analytic sensitivity (lower detection limits) of these tests [36].

## Figures and Tables

**Table 1:** Sensitivity and specificity of the examined SARS-CoV-2 Ag-RDTs in comparison to rRT-PCR. (1) Sensitivity % (PCR-positive samples), (2) specificity % (rRT-PCR-negative samples).

			RIDA^®^QUICK SARS-CoV-2 Antigen (R-Biopharm)	SARS-CoV-2 Rapid Antigen Test (Roche)	NADAL^®^ COVID-19 Ag Test (Nal von Minden)	SARS-CoV-2 Ag Test (LumiraDx)
	rRT-PCR (Target Gene)		ORF1			
(1) Sensitivity	rRT-PCR positive all samples	*n* = 74	39.2% (29/74) (28–51.2% 95% CI)	43.2% (32/74) (37.8–55.3% 95% CI)	24.3% (18/74) (15.1–35.7% 95% CI)	50% (37/74) (38.1–61.9% 95% CI)
	≥6 log_10_ RNA copies/mL	*n* = 21	85.7% (18/21)	100% (21/21)	76.2% (16/21)	100% (21/21)
(2) Specificity	rRT-PCR negative	*n* = 26	96.2% (25/26) (80.4–99.9% 95% CI)	100% (26/26) (86.8–100% 95% CI)	100% (26/26) (86.8–100% 95% CI)	100% (26/26) (86.8–100% 95% CI)

**Table 2:** Positive test probability (50% and 95%) for the examined Ag-RDTs.

Ag-RDT	Positive Test with 50% Probability (Values in log_10_ RNA/mL)	Positive Test with 95% Probability (Values in log_10_ RNA/mL)
R-Biopharm	5.43 (4.997–5.858)	7.415 (6.499–8.475)
Roche	5.196 (4.869–5.54)	6.564 (5.874–7.239)
Nal von Minden	6.099 (5.778–6.427)	7.263 (6.631–7.935)
LumiraDx	4.875 (4.578–5.169)	5.979 (5.41–6.539)

Values in columns denote the log_10_ RNA/mL concentration at which the test returns a positive result with 50% and 95% probability, respectively, according to a Bayesian logistic regression fitted to the data. Point estimates are the maximum a posteriori estimates, and their confidence bands in parentheses are the upper and lower bounds of the 95% highest posterior probability density.

**Table 3:** Sensitivity and specificity of the examined SARS-CoV-2 Ag-RDTs. (1) Sensitivity % (cell culture-positive samples), (2) specificity % (cell culture-negative samples).

Cell Culture		RIDA^®^QUICK SARS-CoV-2 Antigen (R-Biopharm)	SARS-CoV-2 Rapid Antigen Test (Roche)	NADAL^®^ COVID-19 Ag Test (Nal von Minden)	SARS-CoV-2 Ag Test (LumiraDx)
(1) Sensitivity	*n* = 34	61.8% (21/34) (43.6–77.8% 95% CI)	70.6% (24/34) (52.5–84.9% 95% CI)	50% (17/34) (32.4–67.6% 95% CI)	82.4% (28/34) (65.5–93.2% 95% CI)
(2) Specificity	*n* = 31	93.6% (29/31) (78.6–99.2% 95% CI)	77.4% (24/31) (58.9–90.4% 95% CI)	96.8% (30/31) (83.3–99.9% 95% CI)	77.4% (24/31) (58.9–90.4% 95% CI)

**Table 4:** Cohen’s weighted kappa coefficient between the Ag-RDTs and the cell culture results.

	RIDA^®^QUICK SARS-CoV-2 Antigen (R-Biopharm)	SARS-CoV-2 Rapid Antigen Test (Roche)	NADAL^®^ COVID-19 Ag Test (Nal von Minden)	SARS-CoV-2 Ag Test (LumiraDx)
weighted kappa	0.545	0.478	0.457	0.599
standard error	0.097	0.108	0.095	0.099
95% CI	0.354–0.735	0.266–0.690	0.270–0.644	0.404–0.794

Kappa < 0.20: poor agreement; 0.21–0.40: fair agreement; 0.41–0.60: moderate agreement; 0.61–0.80: substantial agreement; 0.81–1.00: almost perfect agreement.

## Data Availability

Data is contained within the supplementary material.

## Supplementary Materials

The following are available online at https://www.mdpi.com/2077-0383/10/2/328/s1: Figure S1: Generated standard curve for the ORF1 gene using the quantitative SARS-CoV-2 RNA comparison samples. Figure S2: Graphical display of Bayesian logistic regression models fitted to the antigen test results. Table S1: rRT-PCR CT values of tested SARS-CoV-2 quantitative comparison samples. Table S2: Used SARS-CoV-2 Ag-RDT sample volumes and reagent amounts according to the manufacturers’ and study’s protocol. Table S3: SARS-CoV-2 rRT-PCR, SARS-CoV-2 Ag-RDTs and cell culture results for the examined clinical samples. Tables S4–S7: Cohen’s weighted kappa coefficient between the R-Biopharm, Roche, Nal von Minden, or LumiraDx test, respectively, and cell culture results.
