# Supplementary material for: The Comparative Clinical Performance of Four SARS-CoV-2 Rapid Antigen Tests and Their Correlation to Infectivity In Vitro
Source: J Clin Med. 2021 Jan 17;10(2):328. doi: 10.3390/jcm10020328 (PMC7830733; doi:10.3390/jcm10020328)
Supplement: Supplementary file 1 [file jcm-10-00328-s001.pdf]

Supplementary Material

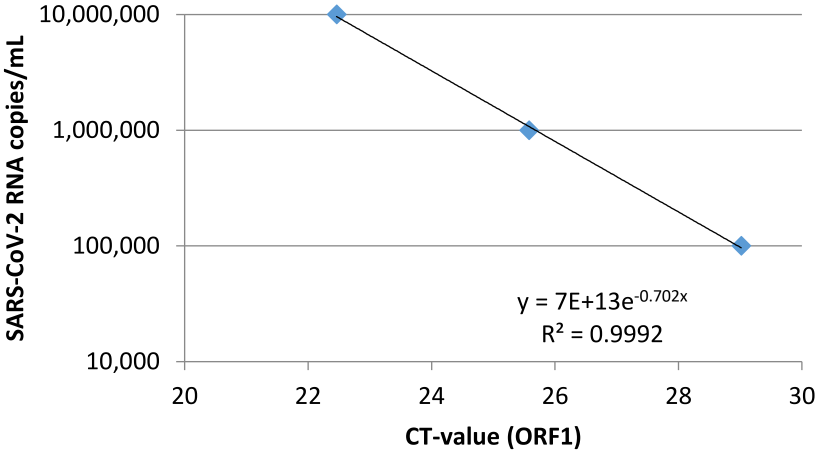

Figure S1 – Generated standard curve for the ORF1 gene using the quantitative SARS-CoV-2 RNA comparison samples.

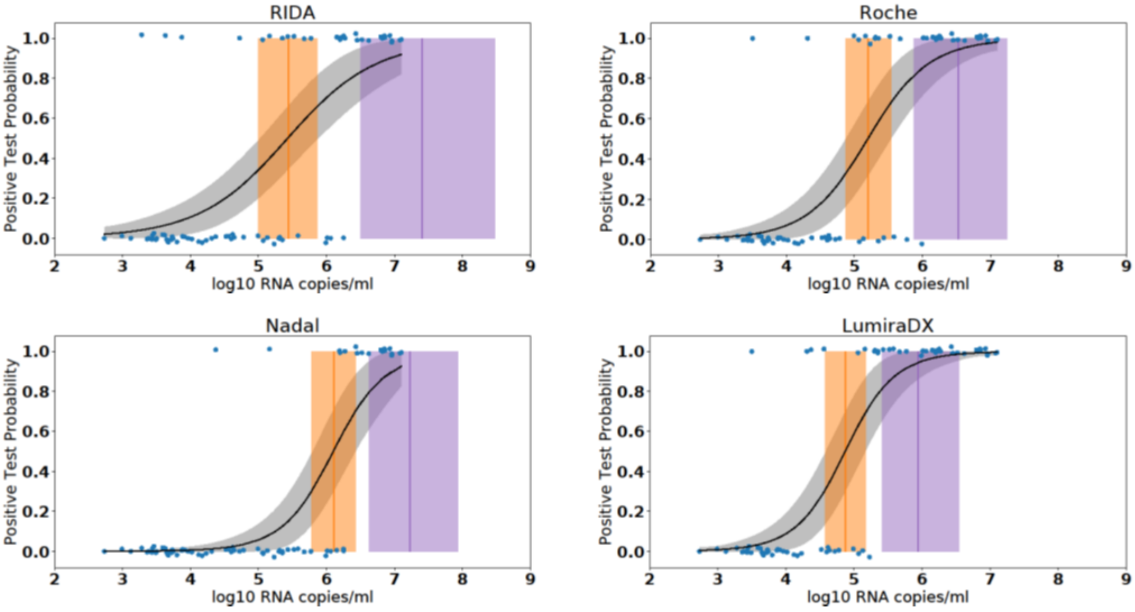

Figure S2 - Graphical display of Bayesian logistic regression models fitted to the antigen test results, with random vertical displacement of samples for a better sense of quantity. The black line is the mean probability of a positive test result at a given concentration from the fitted model. It is surrounded by grey bands representing the associated 95% highest posterior density interval. The vertical orange and purple lines represent decision boundaries for the concentration at which the test returns a positive result with 50% and 95% probability, respectively (see Table SX). They are each surrounded by 95% highest posterior density interval bands. Antigen tests used for this analysis: RIDA (R-Biopharm), Roche, Nadal (nal von minden), LumiraDx.

Table S1 – RT-PCR CT values of tested SARS-CoV-2 quantitative comparison samples.

Comparison sample 1	Comparison sample 2	Comparison sample 3
5 log_{10} SARS-CoV-2 RNA copies/mL	6 log_{10} SARS-CoV-2 RNA copies/mL	7 log_{10} SARS-CoV-2 RNA copies/mL

	CT value (ORF1)		
Day 1	28.96	25.4	22.37
	28.9	25.07	21.91
	29	25.36	22.01
	28.82	25.44	22.52
	28.84	25.4	22.16
Day 2	29.15	25.9	22.57
	29.32	25.84	23.01
	29.01	25.84	22.85
	28.72	25.93	22.64
	29.44	25.59	22.57

Table S2 – Used SARS-CoV-2 Ag-RDT sample volumes and reagent amounts according to the manufacturers' and study's protocol.

SARS-CoV-2 Ag-RDT	Manufacturer's protocol	Study protocol
RIDA®QUICK SARS-CoV-2 Antigen (R-Biopharm)	2 drops out of the specimen vial with<br>3 drops reagent A & B (ca. 50 µL each)<br>-> reagent vial with test dip stick	50 µL of specimen swab in PBS (2 mL) with<br>50 µL reagent A & B (each)<br>-> reagent vial with test dip stick
SARS-CoV-2 Rapid Antigen Test (Roche)	specimen swab in extraction vial with<br>10 drops reagent (ca. 350 µL extraction buffer<br>-> 3 drops on test cassette	100 µL of specimen swab in PBS (2 mL) with<br>100 µL reagent<br>-> 3 drops on test cassette
NADAL® COVID-19 Ag Test (nal von minden)	specimen swab in extraction vial with<br>10 drops reagent (ca. 350 µL extraction buffer), remove swab<br>-> 2 drops on test cassette	100 µL of specimen swab in PBS (2 mL) with<br>100 µL reagent<br>-> 2 drops on test cassette
SARS-CoV-2 Ag Test (LumiraDx)	specimen swab in extraction vial including reagent (ca. 700 µL extraction buffer)<br>-> 1 drop on test strip	100 µL of specimen swab in PBS (2 mL) with<br>100 µL reagent<br>-> 1 drop on test strip

Incubation periods, hands-on steps and readout times were performed according to the manufacturer's protocol.

Table S3 – SARS-CoV-2 RT-PCR, SARS-CoV-2 Ag-RDTs and cell culture results for the examined clinical samples.

Sample Nr.	SARS-CoV-2 RT-PCR				RIDA®QUICK SARS-CoV-2 Antigen (R-Biopharm)	SARS-CoV-2 Rapid Antigen Test (Roche)	NADAL® COVID-19 Ag Test (nal von minden)	SARS-CoV-2 Ag Test (LumiraDx)	Cell culture
	ORF1 gene	ORF1 gene log_{10} RNA copies/mL	E gene	E gene log_{10} RNA copies/mL					

	(CT-value)		(CT-value)						
1	22.2	7.08	22.11	6.86	pos.	pos.	pos.	pos.	pos.
2	22.13	7.1	22.27	6.81	pos.	pos.	pos.	pos.	pos.
3	22.94	6.85	22.39	6.78	pos.	pos.	pos.	pos.	pos.
4	22.59	6.96	22.45	6.76	pos.	pos.	pos.	pos.	pos.
5	22.58	6.96	22.82	6.64	pos.	pos.	pos.	pos.	pos.
6	22.68	6.93	22.68	6.69	pos.	pos.	pos.	pos.	pos.
7	23.11	6.8	22.85	6.64	pos.	pos.	pos.	pos.	pos.
8	22.9	6.86	neg.		pos.	pos.	pos.	pos.	pos.
9	23.05	6.82	23.01	6.59	pos.	pos.	pos.	pos.	pos.
10	23.71	6.62	23.62	6.4	pos.	pos.	pos.	pos.	pos.
11	24.26	6.45	23.96	6.3	pos.	pos.	pos.	pos.	pos.
12	24.03	6.52	24.04	6.27	pos.	pos.	pos.	pos.	pos.
13	24.31	6.43	24.17	6.23	pos.	pos.	pos.	pos.	pos.
14	24.84	6.27	24.96	5.99	pos.	pos.	pos.	pos.	neg.
15	25.06	6.2	24.47	6.14	pos.	pos.	pos.	pos.	pos.
16	24.91	6.25	25.07	5.96	neg.	pos.	neg.	pos.	pos.
17	24.91	6.25	25.14	5.94	pos.	pos.	neg.	pos.	pos.
18	25.1	6.19	25.33	5.88	pos.	pos.	pos.	pos.	pos.
19	25.25	6.15	25.12	5.94	pos.	pos.	neg.	pos.	pos.
20	25.55	6.06	neg.		neg.	pos.	neg.	pos.	neg.
21	25.7	6.01	25.75	5.75	neg.	pos.	neg.	pos.	pos.
22	25.77	5.99	neg.		neg.	neg.	neg.	pos.	pos.
23	26.48	5.77	27.15	5.32	pos.	neg.	neg.	pos.	tx
24	26.8	5.67	27.14	5.33	pos.	pos.	neg.	pos.	neg.
25	27.12	5.58	27.23	5.3	neg.	neg.	neg.	pos.	pos.
26	27.3	5.52	27.78	5.13	pos.	pos.	neg.	pos.	pos.
27	27.59	5.43	28.18	5.01	pos.	neg.	neg.	pos.	tx
28	27.85	5.35	27.89	5.1	neg.	neg.	neg.	pos.	pos.
29	27.87	5.35	28.28	4.98	pos.	pos.	neg.	pos.	pos.
30	28.01	5.31	27.99	5.07	neg.	pos.	neg.	pos.	neg.
31	28.04	5.3	28.19	5.01	neg.	pos.	neg.	pos.	neg.
32	28.25	5.23	28.95	4.78	neg.	pos.	neg.	neg.	pos.
33	28.82	5.06	28.43	4.93	pos.	pos.	neg.	pos.	pos.
34	28.48	5.16	28.75	4.84	pos.	pos.	pos.	pos.	pos.
35	28.58	5.13	28.61	4.88	neg.	neg.	neg.	neg.	neg.
36	28.79	5.07	29.29	4.67	neg.	neg.	neg.	neg.	pos.
37	29.05	4.99	29.86	4.5	neg.	pos.	neg.	neg.	neg.
38	29.72	4.78	30.35	4.35	neg.	neg.	neg.	neg.	pos.
39	29.92	4.72	30.11	4.42	pos.	neg.	neg.	neg.	tx
40	30.18	4.64	30.61	4.27	neg.	neg.	neg.	neg.	neg.
41	30.26	4.62	30.77	4.22	neg.	neg.	neg.	neg.	neg.
42	30.32	4.6	30.6	4.27	neg.	neg.	neg.	neg.	pos.
43	30.46	4.56	31.14	4.12	neg.	neg.	neg.	pos.	neg.
44	30.6	4.52	32.45	3.71	neg.	neg.	neg.	neg.	neg.

45	31.08	4.37	31.65	3.95	neg.	neg.	pos.	pos.	pos.
46	31.28	4.31	31.87	3.89	neg.	pos.	neg.	pos.	neg.
47	31.53	4.23	31.94	3.86	neg.	neg.	neg.	neg.	neg.
48	31.72	4.17	32.63	3.65	neg.	neg.	neg.	neg.	neg.
49	31.91	4.12	32.74	3.62	neg.	neg.	neg.	neg.	tx
50	32.23	4.02	33.29	3.45	neg.	neg.	neg.	neg.	neg.
51	32.42	3.96	33.01	3.54	neg.	neg.	neg.	neg.	neg.
52	32.58	3.91	33.5	3.39	neg.	neg.	neg.	neg.	neg.
53	32.68	3.88	34.48	3.09	neg.	neg.	neg.	neg.	neg.
54	32.72	3.87	33.58	3.36	pos.	neg.	neg.	neg.	tx
55	32.94	3.8	34.21	3.17	neg.	neg.	neg.	neg.	neg.
56	33.15	3.74	34.95	2.95	neg.	neg.	neg.	neg.	neg.
57	33.19	3.73	34.64	3.04	neg.	neg.	neg.	neg.	neg.
58	33.22	3.72	34.31	3.14	neg.	neg.	neg.	neg.	neg.
59	33.39	3.67	34.84	2.98	neg.	neg.	neg.	neg.	neg.
60	33.45	3.65	34.41	3.11	neg.	neg.	neg.	neg.	tx
61	33.5	3.63	35.82	2.68	pos.	neg.	neg.	neg.	tx
62	33.53	3.62	35.34	2.83	neg.	neg.	neg.	neg.	neg.
63	33.65	3.59	34.37	3.12	neg.	neg.	neg.	neg.	neg.
64	33.92	3.5	35.34	2.83	neg.	pos.	neg.	pos.	pos.
65	33.96	3.49	34.78	3	neg.	neg.	neg.	neg.	tx
66	34.07	3.46	34.68	3.03	neg.	neg.	neg.	neg.	neg.
67	34.13	3.44	37.68	2.11	neg.	neg.	neg.	neg.	neg.
68	34.16	3.43	35.54	2.77	neg.	neg.	neg.	neg.	neg.
69	34.32	3.38	36.29	5.59	neg.	neg.	neg.	neg.	neg.
70	34.4	3.36	35.36	2.82	neg.	neg.	neg.	neg.	neg.
71	34.66	3.28	36.26	2.55	pos.	neg.	neg.	neg.	tx
72	35.18	3.12	35.79	2.69	neg.	neg.	neg.	neg.	tx
73	35.61	2.99	36.93	2.34	neg.	neg.	neg.	neg.	pos.
74	36.46	2.73	neg.		neg.	neg.	neg.	neg.	neg.
75	neg.		36.82	2.38	neg.	neg.	neg.	neg.	pos.
76	neg.		38.69	1.81	neg.	neg.	neg.	neg.	tx
77	neg.		neg.		pos.	neg.	neg.	neg.	-
78	neg.		neg.		neg.	neg.	neg.	neg.	-
79	neg.		neg.		neg.	neg.	neg.	neg.	-
80	neg.		neg.		neg.	neg.	neg.	neg.	-
81	neg.		neg.		neg.	neg.	neg.	neg.	-
82	neg.		neg.		neg.	neg.	neg.	neg.	-
83	neg.		neg.		neg.	neg.	neg.	neg.	-
84	neg.		neg.		neg.	neg.	neg.	neg.	-
85	neg.		neg.		neg.	neg.	neg.	neg.	-
86	neg.		neg.		neg.	neg.	neg.	neg.	-
87	neg.		neg.		neg.	neg.	neg.	neg.	-
88	neg.		neg.		neg.	neg.	neg.	neg.	-
89	neg.		neg.		neg.	neg.	neg.	neg.	-

90	neg.		neg.		neg.	neg.	neg.	neg.	-
91	neg.		neg.		neg.	neg.	neg.	neg.	-
92	neg.		neg.		neg.	neg.	neg.	neg.	-
93	neg.		neg.		neg.	neg.	neg.	neg.	-
94	neg.		neg.		neg.	neg.	neg.	neg.	-
95	neg.		neg.		neg.	neg.	neg.	neg.	-
96	neg.		neg.		neg.	neg.	neg.	neg.	-
97	neg.		neg.		neg.	neg.	neg.	neg.	-
98	neg.		neg.		neg.	neg.	neg.	neg.	-
99	neg.		neg.		neg.	neg.	neg.	neg.	-
100	neg.		neg.		neg.	neg.	neg.	neg.	-

pos. = positive; neg. = negative; tx = cytotoxic; - = not examined

Table S4 – Cohen's weighted kappa coefficient between the R-Biopharm test and cell culture results.

	**RIDA®QUICK SARS-CoV-2 Antigen (R-Biopharm)**		
**Cell culture**	negative	positive	row marginal
negative	29	2	31 (47.7%)
positive	13	21	34 (52.3%)
column marginal	42 (64.6%)	23 (35.4%)	65
weighted kappa	0.545		
standard error	0.097		
95% CI	0.354 – 0.735		

Table S5 – Cohen's weighted kappa coefficient between the Roche test and cell culture results.

	**SARS-CoV-2 Rapid Antigen Test (Roche)**		
**Cell culture**	negative	positive	row marginal
negative	24	7	31 (47.7%)
positive	10	24	34 (52.3%)
column marginal	34 (52.3%)	31 (47.7%)	65
weighted kappa	0.478		
standard error	0.108		
95% CI	0.266 – 0.690		

Table S6 – Cohen's weighted kappa coefficient between the nal von minden test and cell culture results.

	NADAL® COVID-19 Ag Test (nal von minden)		
**Cell culture**	negative	positive	row marginal
negative	30	1	31 (47.7%)
positive	17	17	34 (52.3%)
column marginal	47 (72.3%)	18 (27.7%)	65
weighted kappa	0.457		
standard error	0.095		
95% CI	0.270 – 0.644		

Table S7 – Cohen's weighted kappa coefficient between the LumiraDx test and cell culture results.

	SARS-CoV-2 Ag Test (LumiraDx)		
**Cell culture**	negative	positive	row marginal
negative	24	7	31 (47.7%)
positive	6	28	34 (52.3%)
column marginal	30 (46.2%)	35 (53.8%)	65
weighted kappa	0.599		
standard error	0.099		
95% CI	0.404 – 0.794		
